# Acute forearm compartment syndrome in the setting of acquired hemophilia A

**DOI:** 10.1080/23320885.2022.2071274

**Published:** 2022-05-13

**Authors:** Giscard Joel Adeclat, Monique Hayes, Michael Amick, Joseph Kahan, Andrea Halim

**Affiliations:** Department of Orthopaedics and Rehabilitation, Yale University School of Medicine, New Haven, CT, USA

**Keywords:** Compartment Syndrome, acquired hemophilia, congenital hemophilia, fasciotomy, factor eight inhibitor bypass activity, activated prothrombin complex concentrate

## Abstract

Patients with acquired or congenital hemophilia are at risk for Acute Compartment Syndrome (ACS) and pose a diagnostic challenge and a treatment risk with post-fasciotomy hemostasis of critical importance. We present the case of a woman with ACS of the forearm in the setting of newly diagnosed acquired hemophilia A.

## Introduction

Acute compartment syndrome (ACS) is defined as a critical pressure increase within a confined compartmental space causing a decline in the perfusion pressure to that compartment’s tissues [1]. This may be the result of extrinsic forces such as a tight cast or dressing, or intrinsic forces such as excessive interstitial fluid pressure [2]. These forces can lead to microvascular compromise and decrease the perfusion gradient below a critical value leading to tissue ischemia within that osseofascial compartment [1]. Left untreated, ACS may result in neurovascular compromise causing loss of function of the affected limb, Volkmann ischemic contracture, rhabdomyolysis, limb loss and death [1,6]. Although most cases of ACS are associated with trauma and large-vessel damage, patients with hemophilia are prone to spontaneous or atraumatic bleeding. This places these patients uniquely at risk and as such may become a significant challenge for the hematologist and orthopedic surgeon [1,2]. Most commonly located within the calf and anterior forearm, the insidious onset of bleeding without a clear etiology highlights the diagnostic difficulty of ACS in these patients while decompressive fasciotomy may be complicated by poor hemostatic control [1–3].

Acquired hemophilia A (AHA) is predominately a disease of the elderly (median age 64–78 years) [7]. Although some cases are associated with malignancy (6.4–18.4%), autoimmune disorders such as rheumatoid arthritis (9.4–17.0%), infection, and certain medications, most cases are idiopathic (43.6–51.9%) in nature [4,7]. Rarely, AHA may be associated with pregnancy [4,7]. AHA is characterized by neutralizing IgG autoantibodies that target endogenous Factor VIII of the clotting cascade, resulting in increased bleeding episodes whereas congenital hemophilia occurs due to an inherited deficiency of a coagulation factor [5,7]. Approximately 70% of patients experiencing bleeding will require hemostatic treatment as episodes may be life or limb-threatening [7,8]. Subcutaneous bleeding is most common in AHA, followed by gastrointestinal bleeding, and muscle bleeding (>40%) [7]. Patients with acquired hemophilia usually have no prior history of bleeding and more often have initial presentation of skin or muscle hemorrhage. In contrast, patients with congenital hemophilia often have a history of soft tissue hemorrhage or hemarthrosis [3].

We present the case of a 69-year-old female with acquired hemophilia A (AHA) having developed ACS to her right forearm, necessitating an emergent decompressive fasciotomy with subsequent complete recovery.

## Case summary

**Figure 1.**
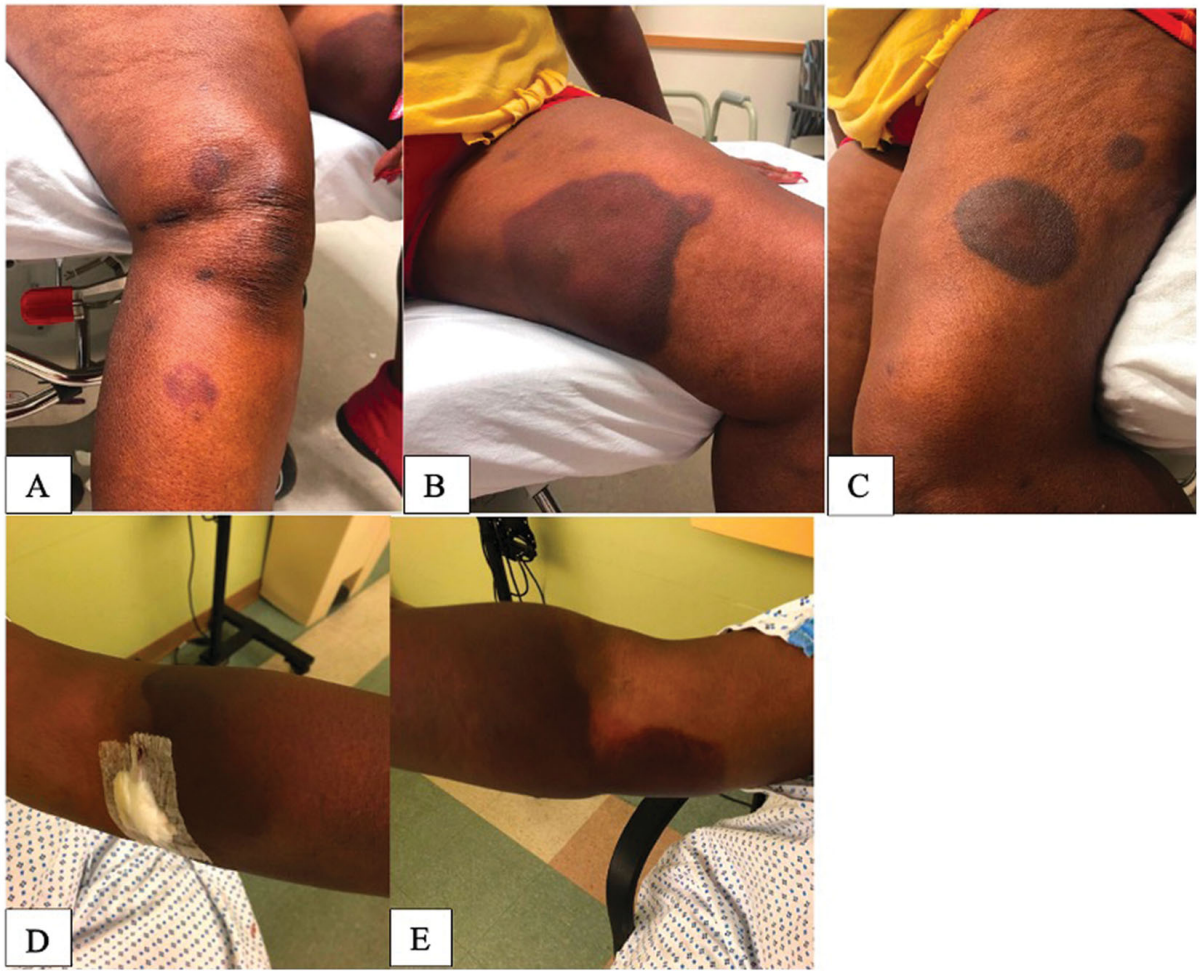
Presentation at diagnosis. (A) Right lower extremity. (B) Left lower extremity (medial thigh). (C) Left lower extremity (outer thigh). (D) Left upper extremity. (E) Right upper extremity.

A 69-year-old female with a history of rheumatoid arthritis originally presented to the emergency department with significant bruising to her bilateral upper and lower extremities after what the patient reported was a painful insect sting or bite to her left forearm 1 week prior (Figure 1(A–E)). Of note, the patient denied any trauma prior to or surrounding this event. Thorough evaluation was performed by the Hematology and the Dermatology service, and it was discovered that the patient had a severe Factor VIII deficiency recorded as 2% (Ref: 66.0–143.0%) likely secondary to the presence of Factor VIII inhibitor. Imaging studies included bilateral upper extremity soft tissue ultrasound that was unremarkable, without evidence of hematoma or drainable fluid collection but rather presence of mild cellulitis. Upon final diagnosis, patient was discharged with a treatment plan to include a 4-week course of Rituximab and Prednisone and subsequent follow up with hematology 3 days post discharge. During this period, the patient initially experienced improvement in swelling and ecchymosis.

Approximately 3-weeks later, the patient presented to her outpatient hematologist with increased right forearm swelling and pain. She was sent to the Emergency Department for further evaluation. In the Emergency Department, it appeared that her symptoms had improved. An examination by the orthopedic surgery team at that time noted that she had mild swelling in her forearm, with no tenderness or pain with passive flexion or extension of the associated wrist and digits. It was felt that there was low concern for compartment syndrome of the right forearm at that time. The patient was discharged in stable condition with outpatient hematology follow up in 1 week.

**Figure 2.**
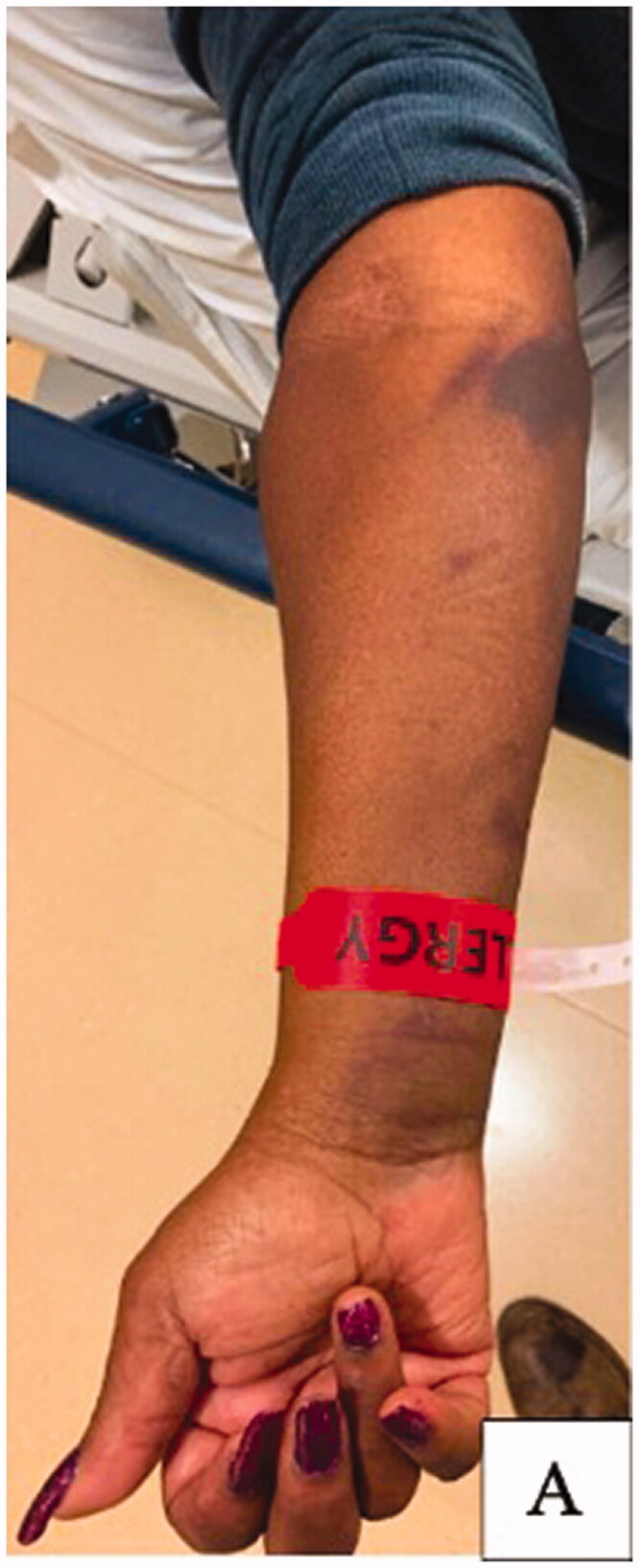
Presentation day of fasciotomy. (A) Right upper extremity with fingers held in flexion.

**Figure 3.**
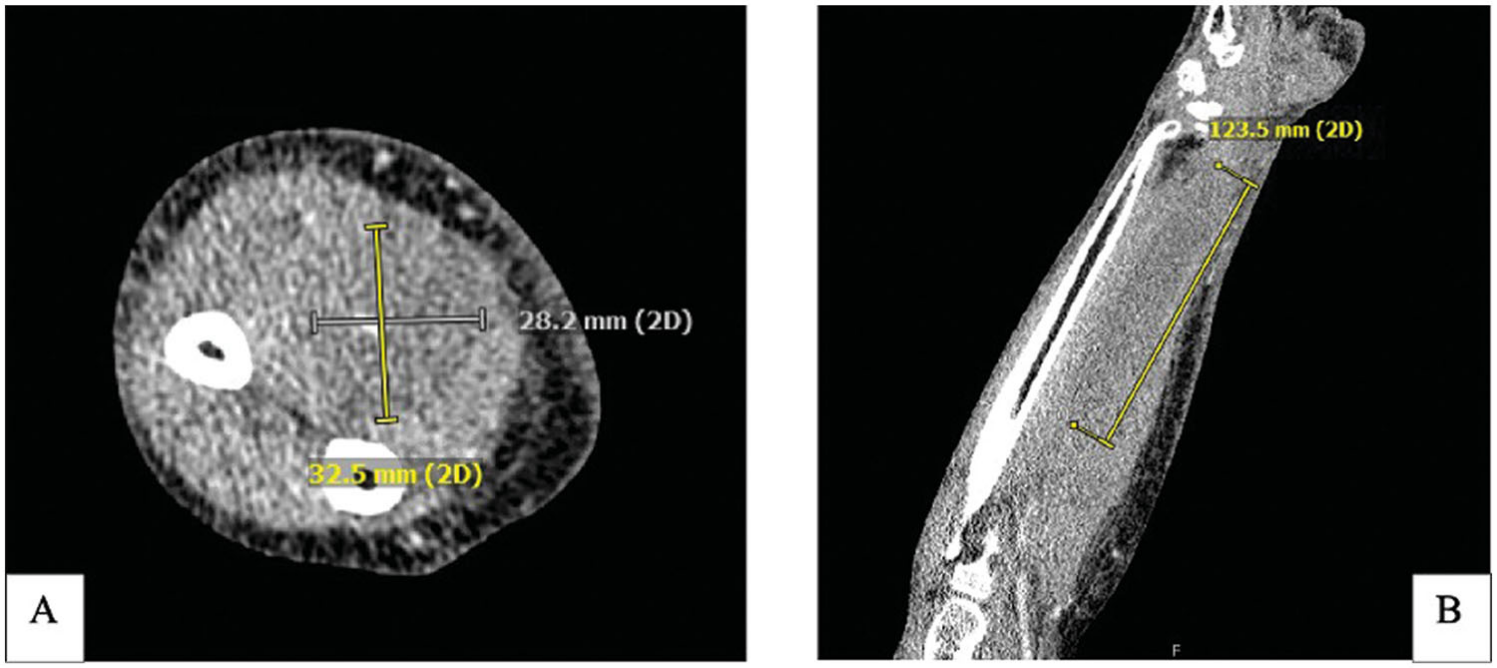
CT Right upper extremity with IV contrast. (A) Axial CT demonstrating a hypodense nonenhancing intramuscular collection within the volar compartment measuring at least 12.3 × 3 × 3 cm. (B) Coronal view of intramuscular collection.

Two-weeks after this encounter, the patient presented to the emergency department with significant pain and swelling to her right forearm that had been progressively worsening over a 1-week period. Examination of the right upper extremity demonstrated ecchymosis to the medial aspect of the elbow with scant ecchymosis along the volar forearm extending to the wrist. There was significant tension in the volar compartment of the forearm with an associated flexion contracture of the 2nd through 5th digits of the right hand (Figure 2(A)). Attempted passive extension of the digits elicited significant pain in the volar compartment. The patient otherwise had normal sensation along the median, ulnar, and radial nerve distribution as well as adequate perfusion of the extremity with a palpable radial pulse. Computed-Tomography with IV contrast of the right upper extremity was performed and demonstrated a 12 × 3 × 3 cm hypodense, non-enhancing intramuscular collection within the volar compartment of the medial forearm consistent with a hematoma (Figure 3(A,B)).

After discussion with hematology, the decision was made to take the patient to the operating room for a right forearm decompressive fasciotomy with evacuation of the deep hematoma. Prior to surgery, hematology recommended and administered the bypassing agent, activated prothrombin complex concentrate (aPCC, FEIBA^®^) both pre- and post-operatively to support hemostasis.

In the operating room, a volar-based forearm incision was made, and the volar compartment fascia was incised with notable muscle bulging from the compartment. Diffuse areas of hematoma were encountered within and surrounding the muscle bellies of the FDP and FDS, primarily in the ulnar forearm. No significant ongoing bleeding was noted, and hemostasis was easily obtained. Both the deep and superficial compartments were fully released. Given the intraoperative findings which were consistent with imaging and exam, we did not feel that the dorsal forearm or hand needed to be additionally fasciotomized. Her skin was easily able to be closed without significant tension, following complete washout and debridement of the hematoma. The patient was placed in a splint for soft tissue rest.

Post operatively, the patient had a remarkable improvement in her hand and forearm symptoms, regaining the ability to both actively and passively extend her fingers without pain. She has remained neurovascularly intact with complete resolution of ecchymoses and swelling. At this time, she continues to undergo treatment for her acquired hemophilia type A (AHA), coordinated by her hematologist.

## Discussion

Compartment syndrome is a rare but potentially limb-threatening condition that must be identified and addressed swiftly [1]. Patients who have hemophilia or other hematologic disorders are at an increased risk of experiencing compartment syndrome due to severe bleeding that can occur following minor stress or trauma [2].

Acute Hemophilia A (AHA) should be suspected in elderly patients with known autoimmune disease and with recent onset of abnormal bleeding in the setting of an isolated prolonged activated partial thromboplastin time (aPTT) and normal prothrombin time (PT) [4,7]. The presence of a clotting factor inhibitor, places these patients at greater risk of orthopedic complications, morbidity, and mortality compared to those with other forms of hemophilia [1,4,5]. We present the case of a patient who developed compartment syndrome of the forearm in the setting of acquired autoantibodies against coagulation factor VIII, also known as acquired hemophilia type A (AHA).

This patient presented initially with bilateral upper and lower extremity bruising after an insect sting to the left forearm, leading to a hematology workup and a diagnosis of acquired factor VIII deficiency. Despite being started on a course of rituximab and prednisone, she continued having intermittent symptoms. Weeks after her initial presentation, she returned with ecchymoses, a flexion contracture, and significant forearm pain concerning for compartment syndrome of the forearm. This required emergent fasciotomy and hematoma evacuation.

There are several reports documenting cases of compartment syndrome in the setting of coagulation factor deficiency [1,2,4]. Interestingly, many of them share a similar time course of symptom onset and feature presentation, including bruising after minor trauma, hematoma formation, and mild symptoms that progress rather insidiously over time. The first paper to ever document compartment syndrome in the setting of hemophilia was published in 1906 [2]. It documented the occurrence of compartment syndrome in two patients with congenital hemophilia following minor trauma to the forearm [2]. Since then, there have been many case reports documenting similar findings with respect to congenital hemophilia, however, the cohort of cases specific to acquired hemophilia is less robust [1,4]. Our case is a valuable addition to the existing literature on this topic, as it provides more context and data for orthopedists to reference should they encounter the rare but potentially devastating case of compartment syndrome in a patient with acquired hemophilia.

In patients with AHA who present with symptoms concerning for early compartment syndrome, it is imperative to decrease compartment pressure and, should they require fasciotomy, obtain hemostatic control. First line hemostatic agents function as either replacement therapy or bypassing therapy [7,8]. True acute compartment syndrome is a surgical emergency and surgical treatment should not be delayed. However if patients with hemophila are suspected to have bleeding within compartments and are promptly treated, decreasing compartment pressure can first be attempted by clotting factor replacement with recombinant porcine factor VIII (rpFVIII) [1,7,8]. In the case of patients with coagulation factor inhibitors, this substitution may not be sufficient and will likely go on to require emergent fasciotomy [1]. As such, control of intra-operative bleeding is critical. Bypassing agents such as Recombinant factor VIIa (rFVIIa) and activated prothrombin complex concentrate (aPCC, FEIBA^®^) should be administered both pre- and post-operatively to correct the underlying factor deficiency and support hemostasis [1,7,8]. Studies have shown indistinguishable efficacy for aPCC compared with rFVIIa, with >90% bleed control rates when used as a first-line treatment [7,8]. Transexamic acid (TXA) in combination with bypassing agents (aPCC or rFVIIa) has been shown to normalize clot stability and case reports on its combination with either agent have provided support for efficacy [7,8]. Further studies are needed, however TXA use could be considered with caution if intra- or post-operative bleeding is not controlled with a bypassing agent alone. In the case of our patient, aPCC was administered pre- and post-operatively without the use of TXA. Intra-operative surgical considerations to control bleeding included use of epinephrine in the local anesthetic to reduce bleeding in the operative field as well as having thrombin-soaked gel foam available should more hemostasis had been required.

It is important for orthopedists and hematologists to be particularly vigilant regarding patients with acquired factor deficiencies, given the inconspicuous physical manifestations that could indicate intermittent bleeding. Patients should also be educated thoroughly about the risks of compartment syndrome and should be made aware of its clinical presentation in the context of hemophilia so that they can seek care in a timely manner.

## Consent

The subject has provided written consent to the inclusion of material pertaining to themselves, that they acknowledge that they cannot be identified *via* the paper; and that they have been have fully anonymized.
